# Development of a Community-Driven Mosquito Surveillance Program for Vectors of La Crosse Virus to Educate, Inform, and Empower a Community

**DOI:** 10.3390/insects13020164

**Published:** 2022-02-03

**Authors:** Rebecca T. Trout Fryxell, Michael Camponovo, Brian Smith, Kurt Butefish, Joshua M. Rosenberg, Julie L. Andsager, Corey A. Day, Micah P. Willis

**Affiliations:** 1 Department of Entomology and Plant Pathology, University of Tennessee, Knoxville, TN 37996, USA; cday11@vols.utk.edu; 2 Department of Geography, University of Tennessee, Knoxville, TN 37996, USA; mcampono@utk.edu; 3 Jefferson Middle School, Oak Ridge Anderson County School District, Oak Ridge, TN 37830, USA; geo4tn@gmail.com; 4 Tennessee Geographic Alliance, University of Tennessee, Knoxville, TN 37996, USA; kbutefis@utk.edu; 5 Department of Education, Health, and Human Sciences, University of Tennessee, Knoxville, TN 37996, USA; jmrosenberg@utk.edu; 6 School of Journalism & Electronic Media, University of Tennessee, Knoxville, TN 37996, USA; jandsage@utk.edu; 7 Department of Agricultural Leadership, Education, and Communications, University of Tennessee, Knoxville, TN 37996, USA; mwill187@vols.utk.edu

**Keywords:** mosquito, surveillance, distribution, zoonoses, collaboration, education, OneHealth, communication, geography

## Abstract

**Simple Summary:**

A pilot program was designed using innovative STEM education programming to create a community-driven surveillance network for mosquitoes that transmit La Crosse virus (LACV). This East Tennessee program is called MEGA:BITESS (Medical Entomology and Geospatial Analyses: Bringing Innovation to Teacher Education and Surveillance Studies). MEGA:BITESS uses student-driven inquiries and classroom learning to engage 6th–12th graders directly with the science of mosquito biology and behaviors and public health science surrounding infectious diseases. As part of the program, the students test hypotheses by collecting surveillance data on mosquitoes carrying LACV, which causes La Crosse encephalitis (LACE), and analyzing their data with an open online platform. Program participants enhance awareness of LACE and help to identify mosquito populations for management, with our long-term goal of reducing LACE cases in children and other vulnerable populations in the region.

**Abstract:**

The fields of entomology, geospatial science, and science communication are understaffed in many areas, resulting in poor community awareness and heightened risks of vector-borne diseases. This is especially true in East Tennessee, where La Crosse encephalitis (LACE) causes pediatric illness each year. In response to these problems, we created a community engagement program that includes a yearlong academy for secondary STEM educators in the 6–12 grade classroom. The objectives of this program were to support inquiry-driven classroom learning to foster student interest in STEM fields, produce community-driven mosquito surveillance, and enhance community awareness of LACE. We trained educators in medical entomology, geospatial science, and science communication, and they incorporated those skills into lesson plans for a mosquito oviposition experiment that tested hypotheses developed in the classroom. Here, we share results from the first two years of the MEGA:BITESS academy, tailored for our community by having students ask questions directly related to *Aedes* mosquito oviposition biology and La Crosse encephalitis. In year one, we recruited 17 educators to participate in the project, and 15 of those educators returned in year two. All participating educators completed the academy, conducted the oviposition experiment, and informed over 400 students about a variety of careers and disciplines for their students. Here, we present a community-based program that helps to address the problems associated with long-term mosquito surveillance, health and science education and communication, career opportunities, and the community needs of Appalachia, as well as the initial data on the effectiveness of two years of an educator-targeted professional-development program.

## 1. Introduction

*Aedes* mosquitoes (Diptera: Culicidae) pose domestic and international threats because of their rapid invasive spread and potential to transmit multiple pathogens of medical and veterinary importance [1]. A unique *Aedes*-related mosquito-borne disease is La Crosse viral disease, neuroinvasive forms cause La Crosse encephalitis (LACE), which is also the leading pediatric arboviral disease in the continental United States [2]. Increasingly recognized since 1997, La Crosse viral disease has been prevalent in southern Appalachia, where approximately 75% of all cases now occur [2,3]. Symptoms vary among individuals, but immunocompromised individuals and children less than 15 years old may present with symptoms similar to commonly associated illnesses (e.g., fever, aches, fatigue, headache) in conjunction with reported mosquito bites [4]. If infection worsens, neurological symptoms may present, including seizures, coma, encephalitis, hemiparesis, paralysis, and/or cognitive disorders [4,5]. An outdated assessment of the economic burden of LACE estimated that the financial costs range from $48,775–$3,090,398 (2003 USD) per case, at an average of $791,374 over 89.6 years of life adjusted for disabilities [6,7]; this equates to $1,199,112 ($73,905–$4,682,658) in 2021.

The causative agent for La Crosse viral disease is the La Crosse virus (LACV), which is transmitted via the bite of a LACV-infected *Aedes* mosquito [8,9]. *Aedes triseriatus* Say is the primary LACV vector, *Ae. albopictus* Skuse is an accessory vector involved with transmission, and *Ae. japonicus* Theobald may also be an accessory vector [10,11,12,13]. All three mosquitoes will oviposit their eggs into similar water-filled artificial and natural habitats (e.g., containers and tree holes), and females will blood feed on humans and sciurid hosts (e.g., squirrels, chipmunks, and groundhogs) [14,15]. Importantly, LACV is maintained in the environment via zoonotic transmission with their sciurid hosts [16] and transovarial transmission from female to offspring [17]. Epidemiological work in southern Appalachia demonstrated that LACE cases are associated with a combination of natural and artificial oviposition sites [15,18,19] and that LACE cases are focal and repetitive at specific sites [20,21], suggesting mosquito management and education is a potential solution to disease prevention. Knowing *Ae. triseriatus* and *Ae. albopictus* are feeding on the same LACV-reservoir hosts, ovipositing in the same natural and artificial environment, and that LACV is focal to specific sites provides us with opportunities to identify and manage these mosquitoes and potentially LACV.

Understanding the temporal and spatial trends of these disease vectors permits the design of targeted mosquito management and control [22]. To reduce the burden of LACV, it is critical to monitor *Aedes* mosquito populations, which includes discovering precisely when and where infected mosquitoes occur in a given region. Fortunately, the surveillance and control of immature and adult *Aedes* spp. has been researched for decades, with techniques specifically developed for *Ae. aegypti* L. and the accessory LACV vector *Ae. albopictus* [23,24,25]. Because LACV vectors are associated with human habitats and share similar ecologies to *Ae. aegypti* and *Ae. albopictus*, standard methods for *Aedes* surveillance can be used for LACV vectors. Like *Ae. aegypti*, LACV vectors can be collected with oviposition containers (~ovitraps) strategically placed in suspected LACV-positive areas, which are typically described as habitats with increased vegetation and many hardwood trees and containers such as cemeteries, parks, forests, and schools [26,27,28,29]. Captured eggs can be reared to adults, and those adults can be screened for LACV [28,30]; the results can be used to identify sites with persistent mosquito populations and LACV infection, which then allows for targeted-mosquito control.

Typically for many arboviruses (e.g., Zika virus, West Nile virus), public health departments apply adulticides to kill adult mosquitoes after a positive human case is identified; a therapeutic and expensive approach for mosquito-borne diseases since mosquito control only occurs after human cases are reported [31]. Unfortunately, the southern Appalachian region has minimal and sporadic mosquito surveillance and management programs. The region’s existing mosquito surveillance infrastructure is limited, primarily dedicated to West Nile virus and concentrated in more urban or metropolitan areas, which does not include the correct surveillance efforts for either LACV or the *Aedes* vectors. Affordable mosquito surveillance and management are dependent upon effective and inexpensive surveillance methods that do not exist; unfortunately, a LACV surveillance program does not exist, thereby creating a public health need to improve LACV and *Aedes* surveillance.

Successful communication of science and health is more multifaceted than most realize due to the complexity of the material, the way it is communicated, and challenges in reaching potentially vulnerable audiences [32]. The source of the information and the delivery method in which the material is presented has an impact on the public’s receptiveness to the information. Health and science communication can be improved when it comes from individuals inside the same community as those receiving the information because they often share the same knowledge, values, and beliefs of that community (NASEM 2017). Multiple reviews and meta-analyses indicate that for mosquito control to be effective, engaging the community in those efforts is necessary [23,24,25]. Additionally, effective science communication on these important issues can build, maintain, and/or restore trust, but efforts must be planned and intentional to be effective [33,34]. To our knowledge, there are no extant public health campaigns against LACV, and most U.S.-based mosquito campaigns target West Nile virus transmitted by *Culex* mosquitoes [35,36] or *Ae. aegypti*-associated diseases such as Zika [37]. Thus, there is an additional public health need to create a LACV campaign that also improves health and science communication specifically for LACE to minimize cases and a campaign developed by members of the community for the community would likely be well received and potentially adopted.

The people who identify and develop mosquito and LACV surveillance, management, and informational campaigns have a plethora of job titles and skill sets. Some are medical entomologists who study mosquito vectors, pathogen transmission cycles, and disease ecology. Most public health departments employ a diverse set of trained individuals who may have limited entomology training but often specialize in other disciplines (e.g., epidemiology, environmental health, data management, toxicology, policy, education, geospatial technologies, and health and science communication). Unfortunately, entomology, geoscience, and science communication careers are currently understaffed, which leads to less monitoring and longer response times to problems, putting our human and animal health and food security at risk [32,38,39,40]. Thus, there is an additional need to increase the awareness of these many disciplines and sciences and to develop a workforce with the desire to pursue these fields. In the absence of this workforce, the already understaffed fields will continue to have decreased surveillance and increased response times for pathogens, putting human and animal health and food security at further risk.

Here, we present a potential solution to the above problems (long-term surveillance, health and science communication, and career awareness) and the health and community needs of Appalachia (*Aedes* surveillance for La Crosse virus). Herein are initial data on the first two years of an educator-targeted professional-development program. Known as the MEGA:BITESS academy, this year-long service-learning engagement program was designed to stimulate innovative classroom teaching and learning, facilitate a workforce interested in entomology, geospatial sciences and science/health communication, and foster a community aware of LACV in East Tennessee. MEGA:BITESS stands for Medical Entomology and Geospatial Analyses: Bringing Innovation to Teacher Education and Surveillance Studies. We trained educators in medical entomology, geospatial analysis, and science communication. The educators then used their skills to develop STEM projects for their middle and high school students that examined environmental factors related to mosquito surveillance. Lesson plans, data, and material presented within can be used as a template to develop a community-driven mosquito surveillance program that enhances community awareness of mosquito-borne diseases. The products of MEGA:BITESS include materials that can be used to inform students of a diverse set of career opportunities and informational materials for local health departments and school systems, and lesson plans for teachers to use in the classroom. Simultaneously, MEGA:BITESS produces spatial-temporal data on *Aedes* populations, fosters community awareness of risks related to mosquitoes and LACE, and promotes high-impact STEM learning for students.

## 2. Materials and Methods

The academy was designed to increase educators’ understanding of the very different disciplines and the material necessary to develop lesson plans and implement those lessons. All components were recorded and posted to our YouTube channel (https://www.youtube.com/channel/UClFstQiji-s6XpZ1qOdvp2A) (accessed on 29 December 2021) and project website (www.megabitess.org) (accessed on 29 December 2021) to provide access to those not participating in-person and for academy participants to use either in class or for curriculum/lesson plan development.

Recruiting was targeted at educators (grades 6–12) in East Tennessee, representing the 30 at-risk counties for LACE. We used an extensive professional network developed by the Tennessee Geographic Alliance to recruit educators with assistance from the Tennessee STEM Innovation Network (totaling 109 school districts working in every county in the state), Tennessee Science Teachers Association (representing teachers in all counties in the state), Tennessee Association of Independent Schools, East TN STEM Hub (serving Knox County and 12 surrounding counties), the UTK Center for Enhancing Education in Mathematics and Sciences, Oak Ridge Associated Universities, and other partners. To recruit, we actively participated in community learning events and created infographics and videos advertising our program.

The project team prioritized applications from sixth-grade science and high school biology educators because the project content aligns with state standards and also benefits those students. A short survey response to questions was a part of the application and included specific questions on how the educators will incorporate the content and mosquito collection project into their courses during the coming academic year. A letter of endorsement and support was required from the applicant’s principal to confirm commitment from and cooperation of the school administration.

To improve retention of participants, a stipend, Professional Continuing Education Units (CEUs), and all required materials were offered to all educators. Materials such as PowerPoints, curated videos, and physical specimens were provided to the educators to enhance and/or supplement classroom learning. For more conceptually difficult lessons, such as experimental design and hypothesis testing, we built how-to videos by editing them in Adobe Premiere Pro (Adobe Systems) and creating effects and transitions in After Effects (Adobe Systems), or by creating animated videos with VideoScribe (Sparkol Limited). These educational materials are also available on our website. Recognizing the difficulty of the 2020 academic year, we also printed and provided masks with our logo for each educator and printed removable stickers for students to place on their school-provided laptops; we hoped this would build a sense of pride and community during a difficult period. A $1000 stipend was provided to educators who completed specific tasks outside of the workshops, which consisted of $400 for completing the mosquito surveillance with their students and $200 per developed lesson plan (one per workshop). The UT Center for Professional Education provided CEUs to recognize and record satisfactory participation in this educator professional development program. One CEU was awarded for each 10 contact hours of workshop participation (a total of 5 CEUs were provided if requested).

Three in-service workshops were developed by the project team: a 5-day introduction workshop (that was held during National Mosquito Control Awareness Week around 24 June), a 1-day GIS/data analysis workshop in February, and a 1-day communication workshop held in April around World Malaria Day (25 April). Two of the workshops were held in-person during most of year one (communication workshop was online April 2020) and completely online in year two (2020–2021) with the same educators, but designed so educators could use the developed curriculum with their students upon return from extended breaks (summer, winter, and spring). STEM career opportunities were enhanced during each workshop by professionals in areas related to MEGA:BITESS topics. These experts spoke of how they became involved in their career specialties and presented emerging research and information on such careers.

The first 5-day training workshop was held during the summer and consisted of mosquito, GIS, and science communication lectures. In year one, surveillance-focused field trips and laboratory tours were also conducted; this did not happen in the second year due to COVID-19. The surveillance training included educator-initiated surveillance around the University of Tennessee agriculture campus, collecting data while georeferencing sites, and counting eggs.

The second in-service workshop focused on using analytical techniques, specifically geospatial analyses using ArcGIS Online (ESRI, Redlands, CA, USA) and analytical techniques using the Common Online Data Analysis Platform [41]; educators could compare their own egg and adult mosquito data as well as data obtained by others. This one day of instruction guided educators and students through the phases of asking and answering geographic and analytical questions. The geo-inquiry process [42,43] and inquiry-driven learning process [44] are similar in guiding educators and their students into thinking like a scientist to explore the world and emphasizing how educators who participate in this project will develop resource materials for their classrooms to guide their students in learning about LACV and how to collect data for the project.

The third in-service workshop was designed to build on the science communication lesson presented during the first 5-day workshop but focused on science and health messaging for specific audiences. Educators learned how to create effective digital and print communication material based on risk communication theories with a target audience in mind. Specifically, the workshop used informational graphics created in Canva (www.canva.com) (accessed 29 December 2021) to disseminate information. Participants explored layout and design to help them understand how to create effective communication material. The workshop focused on message development concepts discussed in the first 5-day workshop to help participants further understand message development and show them how to incorporate their messages into communication material. This workshop was grounded using literature that has explored effective issues and visual communication [45].

We evaluated the workshops in several ways. One primary way was through the use of self-report surveys carried out through the Qualtrics survey platform. We created three types of surveys: a pre- and post-summer workshop survey, very brief surveys (“exit tickets”) that followed each of the days of the summer workshop, and a survey on the effectiveness of the workshops. In this study, we report on the results from the use of the third of these three types of surveys, those on the effectiveness of the workshops. To use these surveys, we applied for and received Institutional Review Board approval to use educators’ responses for research.

Specifically, we administered four effectiveness surveys to participating educators, one each after each of the Spring 2020 data analysis/GIS workshops, the Summer 2020 workshop, the Spring 2021 data analysis/GIS workshop, and the Spring 2021 science communications workshop. Based on their importance to our aims of providing meaningful, relevant, and useful experiences to participating educators, we focused on three questions that were asked in all of the effectiveness surveys on educators’ evaluations of (a) their overall satisfaction with the workshop, (b) the workshop’s relevance to their teaching, and (c) the extent to which the workshop addressed a teaching-related need they experienced. Though these questions could tap teachers’ pedagogical or content knowledge, we did not specify in the questions whether we were interested in either the relevance of the workshop or the extent to which the workshop addressed a teaching-related need that was pedagogical or content-related in nature. Therefore, teachers may have thought of both of these elements of their work together, and future research may lend insights into whether the workshop was more beneficial in terms of bolstering pedagogical or content-related knowledge—or both.

During the summer workshop, each educator learned how to design an experiment around a single question focused on *Aedes* oviposition and how to test that question using experimental design and hypotheses testing. At the beginning of their school year, educators worked with their students to ask and test a hypothesis-driven question concerning oviposition; in other words, each educator and their students identified a testable hypothesis based on habitat and then tested their hypothesis on their school property by placing an equal number of oviposition traps (ovitraps) at sites representing self-identified treatment types. Educators and their students placed ten ovitraps at each school (5 ovitraps per treatment) for 10 weeks (August–October 2019) in year one. In year two, more standardization tests were implemented based on feedback, and all educators set 6 ovitraps (3 ovitraps per treatment) at their campus from 2020 calendar weeks 35–40 (August–October 2020). One educator set 12 ovitraps (3 ovitraps per treatment) during the same period. Materials for ovitraps were provided and included 750 mL black plastic cups (Discount Mugs, Miami, FL, USA), a camping stake to keep the ovitrap in place, seed germination paper (10.2 cm in width; SD3815L, Anchor Paper, Plymouth, MN, USA), envelopes to store the egg papers, and bovine liver powder (#02900396 MP Biomedicals, Solon, OH, USA) to make the infusion (2.5 gallons of dechlorinated water mixed with a half teaspoon of bovine liver powder and stored with the lid on for 72 h) in a provided 5-gallon plastic bucket [28]. All educators were given the same material to run their experiments, and the only known differences were the initial water source for the water infusion, trap placement based on the class’s study design, and random error caused by each educator/student. With their students, educators made infusion water, set and stored ovitraps and egg papers, and georeferenced their sites using Survey123 (ESRI, Redlands, CA, USA). Each educator–student team collected egg papers and replaced the egg paper and 500 mL of infusion.

Once the surveillance period was completed (10 weeks in year 1 and 6 weeks in year 2), egg papers were collected from educators. UT undergraduate and graduate students counted the oviposited eggs and recorded the eggs as hatched (head capsule noticeably open) or embryonating (egg was intact and head capsule was closed). Egg papers with eggs were then allowed to hatch in an environment-controlled biosafety laboratory [28]. To hatch the eggs, a liver powder infusion (as described above, but with an additional 1.5 g of yeast) was created and egg papers were submerged with 500 mL of the infusion water in mosquito breeding chambers (BioQuip, Rancho Dominguez, CA, USA). Egg papers were submerged for 24 h and removed for 24 h, with that process being repeated for three submergence periods. On the final submergence, egg papers were left in the water for 48 h before removal. Upon the final removal of egg papers from the infusion, mosquito breeders with no larvae were removed and recorded as no egg hatching. Those with larvae were supplemented ad libitium with fish flakes, and the larvae were reared to adulthood. Mosquito breeders were checked daily for adults, and any eclosed adults were immediately stored in a −20 °C freezer. All adults were then counted and identified with regard to species and sex [46]. If requested, educational material on rearing and diagnostics were provided to the educators and their students, and a virtual field trip to this rearing space was provided in year two. Mosquito results were provided to the educators at the February analytical workshop to help students answer their specific question(s).

These egg collections and recovered adults were the basis of the first community-driven mosquito surveillance program in East Tennessee. Descriptive and comparative statistics were calculated to describe the *Aedes* collections. To determine if *Aedes* surveillance improved over time, the surveillance results of the 15 educators that participated in both years of the program were compared. Oviposition (presence of eggs and number of eggs), hatch rates in the laboratory (presence of larvae), and adult eclosion (presence of an adult, number of species, abundance) were all measured and compared between the first two years of the program. For each educator’s surveillance program, the overall surveillance design, including percent of egg papers returned, mean distance between traps, mean egg-to-adult percentage, and successful use of Survey123 to collect data, were also compared.

The nonparametric paired Wilcoxon Signed Rank test was used to compare the average number of eggs collected each year by the educators. The paired Student’s *t*-test was used to compare the average number of adults that emerged from those same eggs by each educator from 2019–2020. To account for differences in seasonality between the two study years, only data from the calendar weeks of 35–40 was used for both years. Additionally, because of variations in trap placement due to classroom-driven inquiries, only the three cups that yielded the most eggs at each site were included in the statistical analyses. Data visualizations were also produced to test a subset of student-driven hypotheses to demonstrate how educators and students were able to design and test their own scientific inquiries through the mosquito surveillance project.

Products (e.g., infographics, posters, and videos) for educators and their students were developed by the project team and by the professional community. To showcase the diversity of careers and highlight the people in those professions, lunch-and-learns from each workshop were recorded and shared on a YouTube channel and project website. Presenters included professionals in academia, government jobs, and industry representatives with different degrees (Bachelors through Ph.D.) from each discipline. Special care was taken to include professionals with previous experience working with LACE to create a deeper understanding of the community’s needs and health problems.

For the classroom, professionals were surveyed using Twitter and professional networks to identify job titles from the disciplines of medical entomology, geospatial science, and science communication. Three distinct word clouds were then generated using identified job titles that use ‘entomology, ‘geospatial analyses’, and ‘science communication’. Those word clouds were then overlaid with the MEGA:BITESS logo to create a career awareness poster in Adobe InDesign and Adobe Illustrator (Adobe Systems, San Jose, CA, USA). This poster was provided to each educator to display in their physical classroom, which also served as a visual reminder to students about the project.

To enhance awareness of LACE, educators worked with their students to create informational graphics about mosquitoes and LACV and health prevention via hand-drawn illustrations or with Canva. Together, educators and their students worked through the process of developing their message, deciding what information must be shared and the graphics that will illustrate their message.

## 3. Results

### 3.1. Development of the MEGA:BITESS Academy

**Figure 1:**
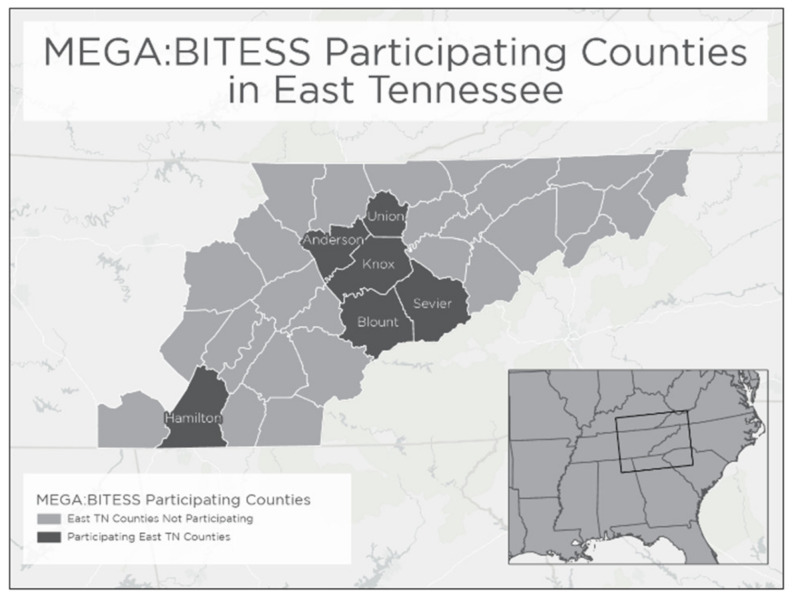
Educators from six counties in eastern Tennessee participated in two years of the program.

In year one of the study, we successfully recruited and trained 17 educators (8 middle school and 9 high school) from 6 counties and 13 schools (Figure 1). In the second year of the project, seven middle school and eight high school educators (6 counties and 12 schools) were retained. Note, some of these educators taught at the same school. The demographics of the educators were 100% Caucasian, 88% female in year one, and 93% female in year two. The two-year academy period began in June 2019 and ended in May 2021. Educator participation was high during the workshop. In the second year of the project, we lost two educators because one moved out of the state and the second had difficulty due to the challenges surrounding teaching during COVID-19. Additionally, during year two, three high school and one middle school educator withdrew from the program, indicating they were having difficulty in managing the work with the pandemic. This resulted in a total of 11 educators who completed two years of the program and more than 415 students (220^+^ 6th–8th graders and 195^+^ 9th–12th graders) participating in the project.

### 3.2. The Effectiveness of the Workshop

As noted above, we focused on three survey questions on the effectiveness of the workshops. Here, we report the results by workshop and overall (Table 1).

The question on the overall effectiveness of the workshop used a 1–5 scale, with 1 indicating “Extremely dissatisfied” and 5 indicating “Extremely satisfied”. These results indicate that educators were—overall—between satisfied and extremely satisfied with the workshops, with some variability in educators’ satisfaction (*M* = 4.29, *SD* = 1.09). Educators’ satisfaction with individual workshops ranged from 3.89 (Spring 2021 Data Analysis/GIS workshop) to 4.70 (Summer 2020 Workshop).

The questions on the relevance of the workshop and the extent to which the workshop addresses a teaching-related need were on a 1–7 scale, with 1 indicating that respondents “strongly disagreed” with the statement about the relevance and extent to which the workshop addresses a need, 4 indicating that respondents neither agreed nor disagreed, and 7 indicating that respondents “strongly agreed” with these statements. Overall, educators reported that they somewhat agreed that the workshop was relevant, again with substantial variation in educators’ experiences (*M* = 5.55, *SD* = 1.45) and that it addressed a teaching-related need (*M* = 5.50, *SD* = 1.29). For both relevance and the extent to which the workshop addresses a need, educators reported that the Summer 2020 Workshop was the most effective, and the Spring 2021 Data Analysis/GIS workshop was the least effective.

### 3.3. Community-Based Mosquito Surveillance

**Figure 2:**
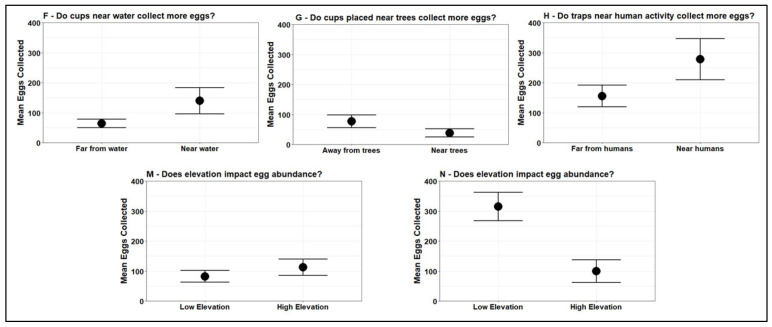
Results of student-driven inquiries from five participating schools in 2020. Schools are anonymously denoted with unique letters (F, G, H, M, and N).

The educators implemented a student-driven mosquito surveillance program with their students in both years of the study. Each educator worked with their students to design an experiment with testable hypotheses, and all educators returned their material both years. Mosquito data for both years are provided in an Open Access database (https://megabitess-tga.hub.arcgis.com/) (accessed 29 December 2021), so educators, the community, and the public can all access and learn about the dataset. We visualized data from a subset of classroom-driven inquiries to provide an example of how students and educators used the mosquito surveillance project to test their own hypotheses (Figure 2).

The surveillance and experimental design of the project improved from year one to year two (Table 2). While fewer ovitraps and egg papers were collected in the second year, the mean distance between ovitraps at schools increased from a mean distance between traps of 24 m to 130 m; this increase in distance between traps occurred for 8 of the 11 schools (73%). Survey123 was used to georeference the ovitrap sites, and 13 schools georeferenced their ovitraps in year one while 12 did in year two; note, 2 schools had 2 educators participating in the project and setting traps.

Of the 1700 egg papers (17 educators × 10 weeks × 10 traps) set in year one, 1626 egg papers were returned (95.65%), and 1120 had eggs on them (68.88%) (Table 2). In total, 102,095 eggs were collected, with a mean of 72.33 eggs per paper. Egg hatching and mosquito rearing resulted in a total of 1214 adult *Ae*. albopictus mosquitoes (mean 0.78 per egg paper), and a maximum of 322 adults per egg paper. In year two, 566 of the potential 576 egg papers (14 educators × 6 weeks × 6 traps; 1 educator × 6 weeks × 12 traps) were returned (Table 2). There was an average of 249 eggs per paper (range: 0–1181), and a total of 71,903 eggs were collected. We hatched the eggs from those collected egg papers and were able to rear two mosquito species: 7826 *Aedes* albopictus and 1802 *Aedes* triseriatus. There was an average of 19 adults reared per egg paper (range: 0–449). The egg to adult percentage was 12.4% (number of adults reared per egg paper/# eggs), which was also better than the previous year (3.41%). Although more egg papers were collected in year one (1700 in 2019 vs. 556 in 2020), more egg papers yielded adults in year two.

**Figure 3:**
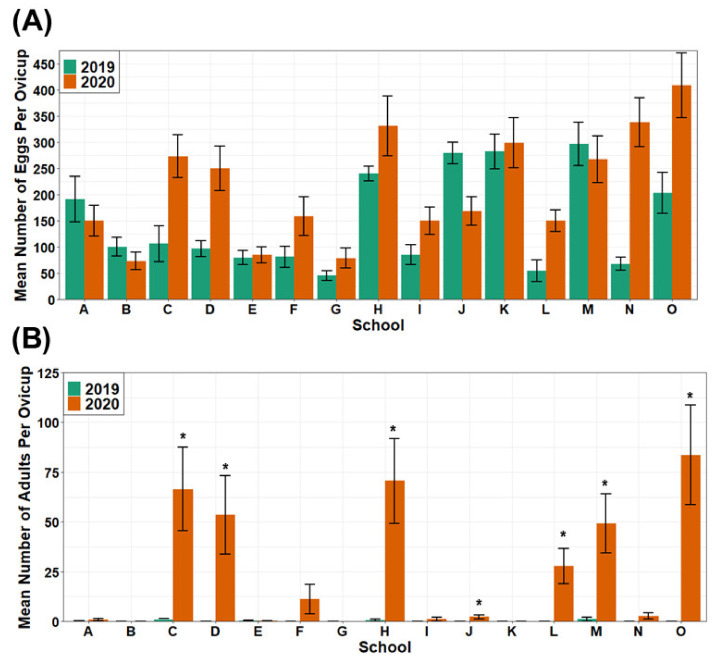
Mean number of eggs (**A**) collected by educators in 2019 and 2020 and average number of adults that emerged from those egg collections (**B**). * = educators stored their egg papers in plastic Tupperware containers in 2020.

Educators (11 of the 15) collected more eggs on average in 2020 than in 2019 (Figure 3A), with an overall significant increase in the number of eggs collected by each educator from 2019 to 2020 (Paired Wilcoxon Signed Rank Test; *p* = 0.04). There was also a significant increase in the number of adults that emerged from those eggs for each educator from 2019 to 2020 (Paired *t*-test; *p* < 0.008), with most of the increase from educators that stored their egg papers in plastic containers during 2020 (Figure 3B).

### 3.4. Increased Awareness of LACV and Career Opportunities

Science communication pieces produced by the students with educators ranged from podcasts and science fair projects to hand-drawn posters and infographics using Canva. Several of the educators created ArcGIS StoryMaps (Esri, Redlands, CA, USA) for their classrooms. The communication workshop featured information on the uses of video and other visuals, social media, and message targeting and distribution in health/science communication. Due to COVID-19, students were limited by distance in the material they produced for health departments.

Additionally, materials were made available online. Since developing the website, we directly engaged with an average of five people per day, ranging from 0 to 43 interactions per day. Interactions with the website included many states within the U.S., but we also had an international reach (Argentina, Cameroon, Canada, China, Egypt, Germany, Ghana, Greece, India, Israel, Malaysia, Nigeria, Taiwan, Thailand, and the United Kingdom). During the 2020 calendar year (1 January–31 December), we had 385 unique visitors. Awareness of both the program and LACV was enhanced by media experts from outside of the project program. Specifically, student groups wrote newspaper articles about their classmates who had been infected with LACV (e.g., https://beardenbark.com/3707/news/environmental-club-ecology-classes-participating-in-utk-mosquito-research/) (accessed 29 December 2021), the Knox County School Board wrote an article about the project at one of the schools and shared it via email to all subscribers (https://www.knoxschools.org/Page/19330?fbclid=IwAR0x2SmKuB4bT3-_BhZhKYR49RW_T1vKB3mtiCCVjJESOOVSZBThCwRvUfo) (accessed 29 December 2021), and the University of Tennessee Institute of Agriculture highlighted the collaborative work between the project team and the educators (https://www.youtube.com/watch?v=kL6wDQImP1I) (accessed 29 December 2021).

## 4. Discussion

The overall objectives of the MEGA:BITESS academy were to create an opportunity for educators and their students to engage in inquiry-driven learning, conduct a community-driven mosquito surveillance program, and enhance the awareness of LACV and career opportunities. Our central hypothesis was that the development of the academy would stimulate innovating classroom teaching and learning, facilitate a workforce interested in entomology and geospatial sciences, and foster a community aware of vector-borne diseases through science communication. Educators participating in the academy incorporated entomology and geospatial sciences in their classrooms throughout the academic year. Some educators wove the material into their lessons, while others developed after-school clubs with their students. Educators and students were able to complete all parts of the study, and educators mentioned that students preferred different parts of the project, suggesting most students were engaged in at least some of the project. Educators mentioned that students who would not normally take a lead role did so in this project; the students in general particularly enjoyed the mosquito surveillance. Other students used creative means of communication to inform their classmates and communities about LACE. Educators became more creative in teaching as they became more confident in the tools and theoretical perspectives employed in the academy. This, in turn, created a small grass-roots community dedicated to LACV and other vector-borne diseases. This community was likely strengthened because of the COVID-19 pandemic, which led to discussions on epidemiology, population curves, and individual and community protection.

During the project, we were met with the challenges of the COVID-19 pandemic as educators in the workshop had to not only learn and use new material but also convert their standard classrooms into flexible-hybrid classes (e.g., in-person and/or virtual). We became flexible and equipped educators with material and training, which also gave them the leadership and the confidence to develop and lead classroom instruction in both learning environments. For example, at a middle school, a Google Classroom (Alphabet/Google, Mountain View, CA, USA) and club were created so students at home and in-person could engage in active learning. Additionally, some of the educators developed lesson plans to be shared with a larger community via our website. We noticed some educators continued to communicate with lunch-and-learn speakers and started interacting with additional scientists in the community to highlight the diversity of careers and people. Another middle school educator developed posters of diverse scientists in a variety of fields for their hallways and then shared those posters with the group. Thus, with the educators, we developed an engaging and relevant in-service academy with a teacher-developed mosquito curriculum and incorporated career paths into the classroom. We are beginning to enhance awareness of agricultural and geospatial career paths, integrate complex concepts into the classroom, and forge mentorships between faculty.

Learning and adapting from year one, educators implemented a student-driven mosquito surveillance program and conducted six weeks of mosquito surveillance on school property with questions designed by students. Each educator worked with their students to design an experiment with testable hypotheses, and all educators returned their material. While mosquitoes were rearing in the BSL-2 room, virtual field trips to the laboratory were offered, and eight classrooms participated. The middle and high school students interacted with the University of Tennessee scientists and asked general questions on mosquito biology, ecology, and rearing while also asking general science/career questions from the students. Using mosquito data collected by the students, we learned that both mosquito vectors are active during the first several weeks of the school year at schools, indicating a mosquito management plan may be necessary at these schools. Collected phenological data corroborated with previously published literature indicating that both mosquito species are active well into October [28,47]. Thus, we enhanced *Aedes* surveillance and began to understand the temporal and spatial models of *Aedes* mosquitoes. More importantly, we developed the groundwork for a community-driven mosquito surveillance program for LACV.

Physical and digital material for educators was an unexpected priority in this project because educators indicated that they were overwhelmed and needed material that could be shared synchronously in the classroom and asynchronously in the virtual classroom. We specifically developed a group of videos for two different audiences. First, tutorials for educators were developed so they could be reminded how to access different material and use it. We also developed material for students so they could understand the experiment and see they were a part of the larger project (https://www.megabitess.org/community-driven-experiment) (accessed 29 December 2021). While LACV-specific health/science communication material on a larger scale was not generated for the East Tennessee community as we had hoped, the material we developed for educators was transferable to the classroom during the pandemic. Educators could engage with their students about virus transmission, epidemiology, diagnostics, disease risk, pathogen prevalence, and the developed material that could transfer to other infectious diseases. We expect that within the next three years students and educators will develop material for their health departments and schools.

The educational component of this project has multiple dimensions. One was that educators and their students assisted with the collection of the aforementioned mosquito-related data that proved valuable from a scientific vantage. Another is that educators and students benefit from opportunities to participate in authentic research experiences. Such experiences are increasingly relevant to and important within K-12 science classrooms as recent reform documents call for all students to not only learn science but to learn to participate in the practices of science [48,49,50]. Specific to scientific data, there are likely several benefits to students having opportunities to “work with”—collect, analyze, and interpret and make sense of—scientific data [48,51,52]. Part of our future research will examine the specific benefits that students experience from participating in research experiences in their classroom as a part of the MEGA:BITESS academy or extensions of it.

Related to students’ experiences are those of educators. We take pride in the positive experiences that educators reported having during the workshops we carried out but also recognize areas that we could improve. Notably, educators’ evaluations of how relevant and useful the academy was (in terms of addressing a teaching-related need they experienced) were lower than their overall satisfaction with the workshops. These issues of relevance and usefulness are key—and are challenges for many professional learning experiences for educators within and beyond K-12 schools and school districts [53]. This is something we plan to enhance in future offerings, including workshops and through the development of other professional learning experiences, such as online learning materials and resources.

This project reports on the first two years of the MEGA:BITESS academy, an in-service academy for educators that allows for community engagement. With educators, a community-driven mosquito and LACV surveillance program was initiated and developed, providing several communities with a system for yearly mosquito assessment and monitoring at schools. Importantly, the students conducting the surveillance were introduced to the scientific method as well as the larger disciplines of entomology, data analysis, epidemiology, geographic sciences, and health/science communications. Knowing mosquito surveillance is largely seasonal, high school graduates and educators could be employed by local health departments as seasonal employees to help with mosquito surveillance as they have already been introduced and trained in many of the techniques.

An exciting utility of the project is the use of these mosquitoes and surveillance data in larger scientific studies. Graduate students can use these mosquitoes to test larger ecological, spatial, temporal, environmental, and genetic questions on *Aedes* mosquitoes and/or LACV. Citizen science projects (e.g., volunteer-based using protocols) and community-engaged science (e.g., participants are collaborators throughout a research process) projects not only provide increased awareness, but they have also generated data used in larger studies and by students in the classroom [54,55]. For example, a citizen science project based out of Texas accepted kissing bugs from the public to assess the distribution, phenology, and *Trypanosoma cruzi* prevalence [56] and similar methods and data on *Ixodes pacificus* and *Ixodes scapularis* and their associated pathogens was uncovered for the U.S. [57,58]. In the field of infectious disease, community-engaged science is strengthened with consistent and clear communication to build relationships, development of contextual knowledge, and adapting over time to improve the project [59]. We fully anticipate that this community-engagement project that incorporates a community-driven mosquito surveillance program can be modified and used by others.

Like other educator and community engagement activities, sustainability for this project will be a continued obstacle [60]. The project’s groundwork took relatively minimal investment ($150,000) and could be maintained with an equal annual investment or significantly expanded with twice the initial investment. We believe this is largely due to the grass-roots and community-inspired project and that this program filled actual classroom and community health and educational needs. Programmatic funding was used for mosquito surveillance (collection, rearing, and LACV testing), to compensate educators for their activities, and to pay for the logistics of the academy. With only a slightly larger investment into community-based surveillance, we know that the program can be continued and expanded to include a more inclusive mosquito surveillance plan (e.g., offering the academy in additional locations outside of Knoxville so educators across the state can participate).

Continual surveillance is critical in understanding mosquito and virus ecology and epidemiology; unfortunately, due to many competing priorities, dedicated resources for this purpose have decreased across numerous states, potentially eroding their ability to quickly and accurately monitor both changes in vector populations and human/animal disease incidence. Nevertheless, based on the success of our passive community-engagement efforts, we believe an investment is worthwhile.

## 5. Conclusions

We developed a community health program to prevent LACV infections in the children of East Tennessee by empowering students, teachers, and the community through STEM education to help control target populations of LACV-infected mosquitoes. The 6th–12th grade STEM education program is grounded in the best practices of recent science education reform initiatives and uses inquiry-driven learning and experiences that open the doors to students’ desire to investigate STEM phenomena. Thus far, 15 trained educators have (1) guided more than 415 students in classroom independent experiments to monitor and assess mosquito populations at their schools and (2) generated high-quality datasets that are being used by University of Tennessee graduate students and will be used by public health professionals. These results will be used to plan and target efficient, cost-effective, and targeted mosquito control efforts. Additionally, students participating in the MEGA:BITESS program gain increased STEM knowledge, with educators reporting students’ high, continuing enthusiasm for and curiosity about the disciplines and subject matter. Students have also engaged in additional communication-based activities at participating schools by creating their own projects, such as submissions to science fair competitions, podcast contests, and school newspaper articles.

## Figures and Tables

**Table 1:** To evaluate each workshop, educators completed surveys and overall, educators were satisfied with each workshop.

Individual Workshops	Number of Complete Participant Responses	Overall Workshop Satisfaction (1–5 Scale)	Workshop Relevance to Educators’ Teaching (1–7 Scale)	Workshop Addresses a Teaching-Related Need (1–7 Scale)
February 2020 Data Analysis/GIS	9	4.12 (*SD* = 1.46)	4.88 (*SD* = 1.89)	4.62 (*SD* = 1.92)
June 2020 Workshop	15	4.70 (0.675)	6.00 (1.25)	6.00 (0.94)
February 2021 Data Analysis/GIS	10	3.89 (1.17)	5.33 (1.50)	5.22 (0.97)
April 2021 Science Communications	8	4.33 (1.05)	5.73 (1.28)	5.80 (1.08)
Overall	42	4.29 (1.09)	5.55 (1.45)	5.50 (1.29)

**Table 2:** Over two years, educators improved with their ability to lead the community-driven mosquito surveillance as indicated by increased adults and increased distance between ovitraps.

Year	Community-Driven Mosquito Surveillance and Decisions								
	No. Papers with Eggs	No. Reared Adults	Mean No. of Eggs (±SE)	No. of Adult Species	Mean No. of Adults (±SE)	Egg Papers Returned (%)	Mean Distance between Ovitraps * (±SE)	Mean Egg-To-Adult % (±SE)	Survey 123
2019	1120 (68.88%)	106 (7.07%)	62.8 (±2.64)	1	0.78 (±0.24)	1626 (95.65%)	26 m (±4.65)	3.41% (±1.13)	13 schools
2020	483 (83.9%)	201 (34.90%)	127 (±6.79)	2	46.97 (±5.00)	566 (98.26%)	75 m (±29.63)	12.4% (±2.28)	12 schools

* 2 schools were removed from this calculation due to changes to their study design as one educator left campus with six traps and a second educator did not use Survey123 to enter their GPS data in year 2.

## Data Availability

All mosquito data are available at the Tennessee Geographic Association Science Hub (https://megabitess-tga.hub.arcgis.com/) (accessed 29 December 2021).
